# The role of macrophage polarization and associated mechanisms in regulating the anti-inflammatory action of acupuncture: a literature review and perspectives

**DOI:** 10.1186/s13020-021-00466-7

**Published:** 2021-07-19

**Authors:** Jiaqi Wang, Shanshan Lu, Fuming Yang, Yi Guo, Zelin Chen, Nannan Yu, Lin Yao, Jin Huang, Wen Fan, Zhifang Xu, Yinan Gong

**Affiliations:** 1 Research Center of Experimental Acupuncture Science, Tianjin University of Traditional Chinese Medicine, No.10 Poyang Lake Road, Tuanbo New Town, Jinghai District, Tianjin, 301617 People’s Republic of China; 2 School of Acupuncture & Moxibustion and Tuina, Tianjin University of Traditional Chinese Medicine, No.10 Poyang Lake Road, Tuanbo New Town, Jinghai District, Tianjin, 301617 People’s Republic of China; 3 School of Traditional Chinese Medicine, Tianjin University of Traditional Chinese Medicine, Tianjin, 301617 People’s Republic of China; 4 Suzuka University of Medical Science, Suzuka, 5100293 Japan; 5 National Clinical Research Center for Chinese Medicine Acupuncture and Moxibustion, Tianjin, 300381 People’s Republic of China

**Keywords:** Acupuncture, Macrophage polarization, Inflammation, HPA axis, Autonomic nerve system, T cells

## Abstract

Acupuncture is used in the treatment of a variety of inflammatory conditions and diseases. However, the mechanisms of its anti-inflammatory action are complex and have not been systematically investigated. Macrophages are key components of the innate immune system, thus, balancing the M1/M2 macrophage ratio and modulating cytokine levels in the inflammatory environment may be desirable therapeutic goals. Evidence has shown that acupuncture has anti-inflammatory actions that affect multiple body systems, including the immune, locomotory, endocrine, nervous, digestive, and respiratory systems, by downregulating pro-inflammatory M1 and upregulating anti-inflammatory M2 macrophages, as well as by modulating associated cytokine secretion. Macrophage polarization is controlled by the interlocking pathways of extrinsic factors, the local tissue microenvironment, and the neural-endocrine-immune systems. It has been suggested that polarization of T lymphocytes and cytokine secretions resulting in modulation of the autonomic nervous system and the hypothalamic–pituitary–adrenal axis, may be upstream mechanisms of acupuncture-induced macrophage polarization. We further propose that macrophage polarization could be the principal pathway involved in acupuncture immune regulation and provide the scientific basis for the clinical application of acupuncture in inflammatory conditions.

## Introduction

Organismal fitness requires an appropriate response to neutralize the threats of infection or injury. Inflammation is a host response defined by the infiltration of immune cells into the affected tissue and plays a key role in mediating the host defense against pathogens, as well as contributing to tissue repair and the recovery of intrinsic homeostasis. As cells of the innate immune system, macrophages are involved not only in the primary response to pathogens, but also in the coordination of the adaptive immune response, inflammation resolution and tissue repair. In response to various stimuli, both resident tissue macrophages (RTMs) and circulating monocyte-derived macrophages alter their basal states in a process known as activation or ‘polarization’. This pleiotropic effect is attributed to different macrophage phenotypes, primarily by differentiation into the classically activated M1 macrophage and the alternately activated M2 macrophage. These subtypes have different transcription spectra and functions [1]. It has been found that the occurrence and development of many inflammatory diseases are closely related to macrophage polarization, and RTMs regulate tissue homeostasis by acting as sentinels with differently polarized macrophages responding to microenvironmental changes, suggesting that balancing the M1/M2 ratio in the inflammatory environment may be a critical therapeutic strategy [2].

Acupuncture is a common complementary and integrative therapy as proved by profuse clinical studies and used by millions of people worldwide. Acupuncture is effective for the treatment of inflammatory diseases and some tissue injuries, such as asthma [3], inflammatory bowel disease [4] and rheumatoid arthritis (RA) [5]. The anti-inflammatory mechanisms of acupuncture are mainly focused on the inhibition of pro-inflammatory factors and the promotion of anti-inflammatory factors. In particular, several recent studies have been demonstrated that acupuncture’s anti-inflammatory action is accompanied by macrophage and T cell polarization.

Here, we initially introduce the crucial roles of macrophage polarization in the pathophysiology of diseases. We then discuss the role of macrophage polarization in acupuncture’s anti-inflammatory action in various systems and diseases. In particular, we discuss the common cellular and molecular mechanisms by which acupuncture regulates macrophage polarization and, in turn, immune function. We propose that macrophage polarization may be the principal pathway involved in acupuncture immune regulation, providing a scientific basis for the clinical application of acupuncture for inflammatory conditions.

## Methods

### Search strategy

Using the PubMed database, we retrieved studies published between January 2011 and March 2021, using medical subject headings and free-text words of [“acupuncture” and “inflammation” or “immune” or “macrophage”] as keywords. The languages were limited to English and Chinese. The filter process was done initially by the website’s search engine retrieved 7998 articles.

### Study selection

**Fig.1:**
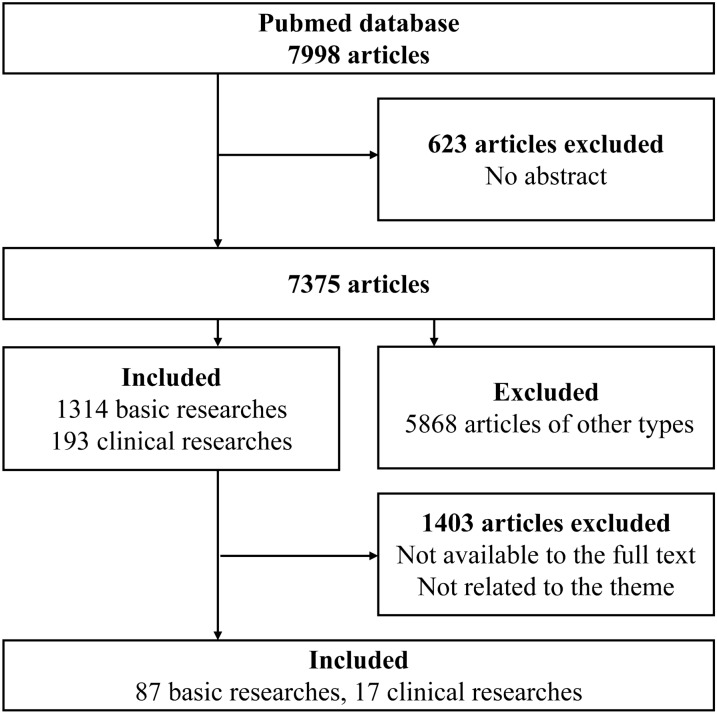
Flow chart of the search strategy and process

Of these articles, 623 articles were excluded due to the absence of abstracts. Of the remaining 7375 articles (including 1314 basic studies and 193 clinical studies) we excluded articles with no full text, and the full texts of the remaining articles were screened manually to exclude those unrelated to the theme of “acupuncture regulating macrophage functions”. Finally, 87 basic studies and 17 clinical studies were included. A flowchart of this search process is shown in Fig. 1.

### Data extraction

The information from 23 typical and recently published studies is listed in Table 1. Data on the study design were extracted and classified using a pre-defined data extraction table, specifying the model type, intervention, acupoints, acupuncture parameters and outcome measurements. Data were extracted by one author and checked by the other authors.

**Table 1:** Regulation of macrophage polarization in the anti-inflammatory action of acupuncture

Refs.	Model/Patient	Intervention	Acupoints	Acupuncture parameters	Improved effect indicators	Mechanisms
Ouyan (2011) [5]	RA patients	EA (n = 34) SN (n = 34)	SN: select 3–5 integral acupoints such as DU20, GB20, LI11, SJ6, RN4, ST36, GB3, GB39, SP6, BL23, BL20, and local Ashi points EA: local Ashi points, BL20 and BL23 for EA, select 3–5 integral acupoints for SN	SN: inserted depth: 0.5–1.5cun, no current, 30 min, once every other day, 30 times EA: continuous wave, adaptable intension, Ashi points for 30 min, BL20 and BL23 for 15 min, other parameters same as SN	–	Peripheral blood and joint synovia EA: TNF-α↓, VEGF↓ SN: TNF-α↓, VEGF↓
Pan (2019) [27]	AIA rats	LA	BL13, KI3	A spot size of 0.2cm^2^, 50 Mw per session for 60 s	Inflammatory mechanical nociception, histopathological changes	Cartilage: TNF-α↓
Torres-Rosas (2014) [30]	Sepsis mice	EA	ST36	10 Hz, 4 V, 40 mA, pulse width: 50 μs, 15 min	–	Serum: TNF-α↓, MCP-1↓, IL-6↓, IFN-γ↓
Shi (2020) [34]	KOA patients	EA (n = 28) MA (n = 30)	ST34, ST35, ST36, EX-LE2, EX-LE4, GB33, SP9, SP10, LR7, LR8, Ashi points, 2–3 distal points including GB31, GB36, GB39, GB41, ST40, ST41, LR3, BL60, SP6, KI3	30 min, 3 sessions per week, 8 weeks EA: 2/100 Hz MA: no current	WOMAC and VAS	Serum EA: TNF-α↓, IL-1β↓, IL-18↓, IL-8↓, MCP-1↓, MMP-3↓, MMP-13↓, IL-13↑ MA: TNF-α↓, IL-1β↓, MMP-3↓, MMP-13↓, IL-13↑
Wu (2019) [35]	KOA rabbits	EA	ST35, EX-LE5	2 Hz/1.1 s and 100 Hz/2.2 s, 17.3 V, pulse width < 1 ms, 30 min, twice a day, 8 weeks	Cartilage morphology	Synovial fluid and cartilage: IL-1β↓, IL-6↓, TNF-α↓, MMP-3↓
Da Silva (2015) [36]	Inflammatory muscle pain mice	MA	SP6	Inserted depth: 2-3 mm, 10 min, once per day, 1, 5, 13 days	Pain behaviors, edema	Gastrocnemius muscle: IL-10↑
Su (2016) [37]	Muscle atrophy mice	Acu-LFES	GB34, ST36	20 Hz, 1 mA,15 min, once per day, 2 weeks	Muscle wasting, muscle regeneration	Muscle: IL-10↑, F4/80 ↑, IL-1β↑, Arg-1↑
Huang (2017) [40]	MCAO/R rats	EA	DU20, DU24	Inserted depth:2-3 mm, 0.2 mA, ≤ 6 V, 2/20 Hz, 30 min per day, 7 days	Neurological deficit, motor and memory impairment	Ipsilateral hippocampus, sensorimotor cortex: IL-1β↓, IL-10↑
Zhao (2017) [42]	SCI rats	EA	GV6, GV9	60 Hz/1.05 s and 2 Hz /2.85 s alternately, ≤ 1 mA, 20 min, every other day, 4 weeks	Basso, Beattie and Bresnahan functional evaluation	Spinal cord: TNF-α↓, IL-1β↓, IL-6↓, IL-10↑, NT-3↑, CD86↓, CD206 ↑
Jiang (2017) [41]	MCAO/R rats	EA	GV20, LI4, LR3	1 mA, 20 Hz /5 min and 2 Hz /30 min	Neurobehavioral, infarct volume	Peri-ischemic cortices: TNF-α↓, IL-1β↓
Kim (2012) [43]	Seizures mice	MA	HT8	Inserted depth:1 mm, once a day, 2 days	Severity of seizure	CA3 of the hippocampus: IL-1β↓, CD11b^+^↓
Jung (2021) [44]	Depression mice	MA	KI10, LR8, LU8, LR4 or non-acupoint	Once a day, 7 days	Depression-like behavior	Spleen: IL-1β↓, COX2↓, TNF-α↓ Liver: IL-1β↓, TNF-α↓
Goes (2014) [52]	Colitis mice	EA	ST36	100 Hz, 1 mA, 20 min, 1 h before and 24 h/ 48 h after the induction of experimental colitis	–	Colon: IL-10↑, iNOS↓
Song (2020) [53]	Irritable bowel syndrome mice	EA	ST25, ST36	Inserted depth: 5 mm, 0.5–1.0 mA, 2/15 Hz, 30 min, once a day, 10 days	Visceral hypersensitivity	Colon: IL-18↓
Yang (2021) [55]	Postoperative ileus mice	EA	ST36	Inserted depth: 3 mm, 1 mA, 10 Hz, plus width: 0.4 ms, 20 min	Intestinal motility	Serum and intestinal: TNF-α↓, Il-6↓, Ach↑
Xue (2014) [57]	Acute pancreatitis rats	EA	ST36	Inserted depth: 5 mm 2-100 Hz, 2 mA, 30 min	Pathological scores of pancreas	Serum: TNF-α↓, IL-6 ↓, Ach↑
da Silva (2011) [56]	Peritonitis mice	MA	SP6	Inserted depth:2-3 mm	Peritoneal leakage	Peritoneal fluid: IL-10↑
Song (2019) [51]	Acute colitis mice	EA	ST36	Inserted depth: 2-3 mm 30 min, HEA: 100 Hz, 1 mA LEA: 10 Hz, 1 mA	Body weight, colon length, DAI score, histological score	Serum HEA and LEA: IL-1β↓, TNF-α↓, IL-6↓, IL-12↓, IL-17↓, NLRP3/IL-1β↓, iNOS↑, Arg-1↑ HEA: IL-10↑, Nrf2/ HO-1↑, CD206↑ LEA: FIZZ1↑
Wei (2015) [61]	Asthma mice	MA	GV14, NBL12, BL13	Inserted depth: 3 mm, 30 min	Airway hyperresponsiveness, mucus secretion	Serum: IgE↓, Th17↓, IL-17A↓, IL-17F↓, IL-22↓ BALF: CD4^+^IL-17A^+^↓, CD4^+^Foxp3^+^↑, lymphocytes↓, eosinophils↓, neutrophils↓ Lung: NF-κBp65↓
Wei (2017) [3]	Asthma mice	MA	GV14, BL12, BL13	Inserted depth: 3 mm, 30 min	Airway hyperresponsiveness	Serum: TNF-α↓, IL-1β↓, IL-5↓ BALF: total leukocyte↓, neutrophil↓, lymphocyte↓ eosinophil↓
Dong (2019) [62]	Asthma mice	MA	GV14, BL12, BL13	Inserted depth: 3 mm, 30 min	Airway hyper-reactivity	Lung: T-bet↑, Foxp3^+^↑, CD4^+^Foxp3^+^↑, IFN-γ^+^↑ Serum: IL-10↑, RORγt↓, CD4^+^IL-17A^+^↓, IL-5↓, IL-13↓, IL-17A↓
Lou (2020) [63]	Lung injury rats	EA	ST36, SP6	Inserted depth: 2-3 mm, dispersed waves: 2/15 Hz, 1 mA, 30 min	Blood gas analysis, lung injury score	Lung: IL-1↓, IL-6↓, TNF-α↓, MPO↓
Li (2020) [64]	Acute respiratory distress syndrome rats	Acupoint catgut embedding	BL13, ST36	Absorbable suture implanted depth: 2-3 mm	Blood gas analysis, lung pressure-volumes, ratio of PaO2/FiO2	BALF: TNF-*α*↓, IL-6↓, IL-10↑

*Acu-LFES* acupuncture plus low-frequency electric stimulation, *BALF* bronchoalveolar lavage fluid, *DAI* disease activity index, *EA* electroacupuncture, *FIZZ1* resistin-likeα, *HEA* high-frequency electroacupuncture, *LA* laser acupuncture, *LEA* low-frequency electroacupuncture, *MA* manual acupuncture, *MPO* myeloperoxidase; *MCAO/R* middle cerebral artery occlusion/reperfusion, *KOA* knee osteoarthritis, *RA* rheumatoid arthritis, *SCI* spinal cord injury, *SN* simple needling, *WOMAC and VAS* the Western Ontario and McMaster Universities Osteoarthritis Index and the Visual Analog Scal

## The crucial role of macrophage polarization in the pathophysiology of disease

In the late nineteenth century, llya Metchnikov described a population of immune cells dedicated to phagocytosis, thus introducing the concept of the macrophage. Macrophages are found in all mammalian tissues, and the most recent view is that most RTMs have an embryonic origin with minimal input from circulating monocytes [6]. With the deepening of the research, different waves of embryonic macrophages have been described, with the first wave deriving from early yolk sac (YS) erythro-myeloid progenitors (EMPs). Primitive macrophages rapidly proliferate and migrate in the embryo [7]. These YS primitive macrophages have been referred to as ‘‘pre-macrophages’’ [8]. Soon afterwards, These EMPs later give rise to YS macrophages, depending on blood to grow in the fetal liver, and giving rise to monocyte-like cells, among other cell types. These monocyte-like cells will thereafter colonize fetal tissues to give rise to fetal monocyte-derived macrophages [9]. Similarly, early hematopoietic stem cells emerging from the aorta-gonad-mesonephros region seed the fetal liver and give rise to myeloid progenitors and monocyte-like cells [10]. Microglia, liver Kupffer cells, lung alveolar macrophages, Langerhans cells and other major RTMs are established before birth and maintain themselves independent of replenishment by blood monocytes during adulthood [11]. Nevertheless, monocytes can infiltrate these tissues in certain circumstances such as in the event of inflammation or when resident macrophages are depleted and are replaced by monocyte-derived macrophages which then proliferate [12]. The intestinal lamina propria and dermal macrophages are constantly replaced from the monocyte population during development [13, 14]. As a result, many RTMs can be of mixed origin [15].

**Fig.2:**
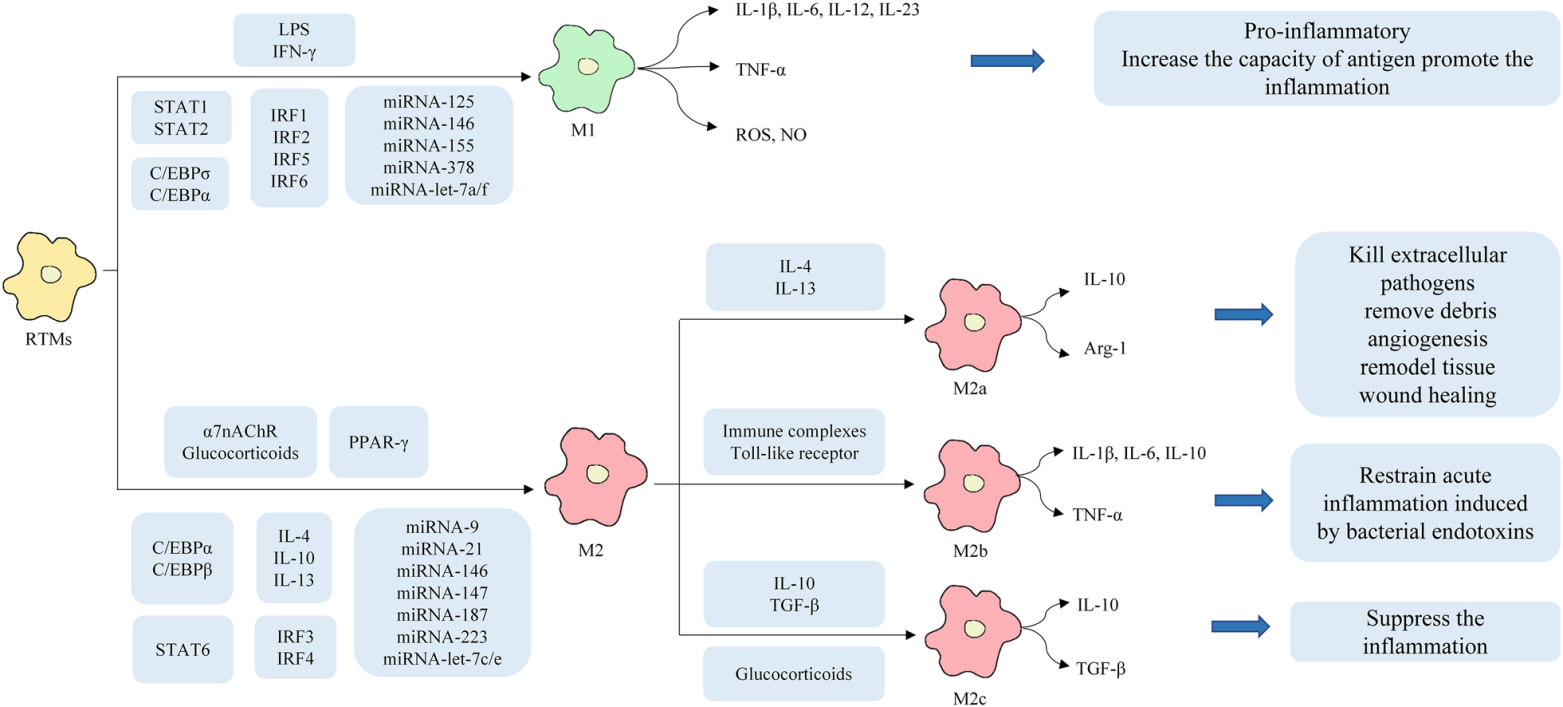
The upstream regulators, phenotypes and functions of macrophage polarization

Depending on different environmental stimuli, macrophages polarize into subtypes that play different roles [16] (Fig. 2). Classically activated macrophages (M1 phenotype) are induced by typical T helper cells (Th) 1 activated by the cytokine interferon-gamma (IFN-γ) or lipopolysaccharide (LPS) and express interleukin (IL)-12^high^, IL-23 ^high^ and IL-10^low^. They mainly secrete pro-inflammatory cytokines, including tumor necrosis factor-α (TNF-α), IL-12, IL-23, IL-1β, IL-6 and chemokines, as well as reactive oxygen species and nitric oxide (NO). Macrophages activated in this way are involved in the acute pro-inflammatory response and have an increased antigen-presenting capacity. In contrast, M2 macrophages generally express IL-12^low^, IL-23^low^ and IL-10^high^, and are usually induced by the Th2 cytokines IL-4 or IL-10 and IL-13. M2 macrophages are classified into three subtypes: M2a, M2b and M2c. M2a macrophages are induced by IL-4 and/or IL-13, secrete the anti-inflammatory cytokine IL-10, and enhance arginase-1 (Arg-1) activity, as well as specifically expressing the mannose receptor (CD206) and macrophage chitinase-like protein 3. They participate in the killing of extracellular pathogens, removal of debris, angiogenesis, tissue remodeling, and wound healing [17]. M2b are induced by immune complexes or the Toll-like receptor, which release high levels of IL-10, TNF-α, IL-6 and IL-1β, restraining acute inflammation induced by bacterial endotoxins and increasing Th2 differentiation and humoral immune responses. M2c macrophages, also known as inactivating macrophages, are induced by IL-10, transforming growth factor-β (TGF-β), and glucocorticoids, and produce large amounts of IL-10 and TGF-β to suppress the inflammation [18–21].

M1 macrophages promote inflammation and are involved in disease progression in a variety of diseases, for example, in certain types of complicated asthma, the action of M1 macrophages results in excessive production of NO that causes oxidative DNA damage, inflammation and aggravated mucus secretion which intensifies lung injury and accelerates airway remodeling [22]. In osteoarthritis (OA), M1 macrophages produce high levels of inflammatory cytokines to exacerbate cartilage degeneration and osteophyte formation, promoting hypertrophic chondrocyte differentiation and maturation, in contrast, M2 macrophages have anti-inflammatory effects, promoting the release of anti-inflammatory factors to reduce inflammation and tissue damage, and stimulating tissue repair [23]. Therefore, the optimal balancing of the M1/M2 ratio and their related molecules in the inflammatory environment to reduce excessive or prolonged M1 polarization and enhance M2 polarization may be desirable therapeutic goals.

## Macrophage polarization in the anti-inflammatory action of acupuncture

The World Health Organization has recommended acupuncture for the treatment of 16 inflammatory diseases such as RA, allergic rhinitis, acute and chronic gastritis, periarthritis of shoulder [24]. According to a nationwide survey of acupuncturists in the USA in 2018, inflammatory diseases accounted for 41% of patients receiving acupuncture treatment [25]. Many studies have shown that regulating macrophage polarization and preventing immune overreaction through endogenous regulatory systems may play key roles in acupuncture’s anti-inflammatory effects in a variety of diseases (Table 1).

### Immune system

RA is one of the most common autoimmune diseases. It is characterized by chronic inflammation of the joints with infiltration of various immune cells, most notably macrophages and T cells, but also neutrophils, mast cells and B cells [26]. Interestingly, most pro-inflammatory cytokines are produced by macrophages, highlighting the important role of macrophages in the pathophysiology of RA. Ouyang et al. [5] found that electroacupuncture (EA) can effectively reduce both TNF-α and vascular endothelial growth factor (VEGF) levels in the peripheral blood and joint synovia, which may be involved in improving the treatment efficacy. Similar results were found in adjuvant-induced arthritic (AIA) rats, where TNF-α levels in the hind ankles were reduced by laser acupuncture at BL60 and KI3 [27]. In our previous work [28], we found that manual acupuncture (MA) at ST36 could alleviate paw edema and pain behavior in AIA rats and mice. Several innate and adaptive immune cytokines were found to be downregulated by MA by day 21. Moreover, bioinformatics analysis showed that the key immune cells for acupuncture regulation in the inflamed ankle joint were monocytes/macrophages. Further study showed that acupuncture may downregulate the numbers of M1 macrophages and the levels of related factors (IL-6, IL-1β, IL-18 and TNF-α) in the inflamed joints and paws. At the same time, MA increased the amount of M2 macrophages and the concentrations of IL-10 and TGF-β, confirming that acupuncture promotes the transition of macrophages from the M1 to the M2 phenotype in inflamed joints, which may be one of the potential key mechanisms of MA anti-inflammatory action [29].

As the precursor cells of macrophages, monocytes also have M1 (pro-inflammatory) and M2 (anti-inflammatory) phenotypes. It has been found that M1 monocytes and their related factors in the blood are dominant in a variety of inflammatory diseases. Studies have shown that EA at ST36 promotes the release of dopamine and reduces the serum levels of multiple M1-induced pro-inflammatory factors induced by LPS-induced sepsis in mice, including TNF-α, monocyte chemoattractant protein-1 (MCP-1) and IL-6 [30]. It was suggested that EA may inhibit M1 polarization of monocytes in the circulation, however, whether EA can alter M1 dominance to M2 dominance in sepsis should be clarified. The above results indicate that acupuncture can regulate the polarization of monocytes and macrophages, which is important in the anti-inflammatory action of acupuncture in immune disorders.

### Locomotor system

OA has a different pathogenesis from the autoimmune RA and is caused by a cartilage differentiation disorder, resulting in pain and dysfunction. Knee osteoarthritis (KOA) is one of the most common diseases which cause significant disability in the world. The findings of Liu et al. [31] showed that the M1/M2 macrophage ratio in the synovial fluids of KOA patients was higher than that in the healthy controls and was positively correlated with KOA severity. It has also been shown that the polarization of M1 macrophages in the synovial fluid can inhibit chondrocyte differentiation [32], and treating with anti-inflammatory cytokines including IL-4, IL-10, or mesenchymal stem cells(MSCs), has been found to inhibit the M1 phenotype and improve ligament and tendon healing [33].

It has been found that eight weeks of treatment with either EA or MA improved the response of KOA patients on both the self-reported pain and function scales, with no difference found between the EA and MA groups. EA and MA also produced a reduction in the serum levels of pro-inflammatory cytokines (TNF-α and IL-1β) and cartilage degradation biomarkers matrix metalloproteinase-3 (MMP-3) and MMP-13, and upregulated levels of the anti-inflammatory cytokine IL-13 [34]. Similar results were observed in KOA rabbits where EA treatment improved cartilage structural arrangement and reduced cellular degeneration, accompanied by reduced levels of IL-1β, IL-6, TNF-α and MMP-3 in the synovial fluid. In addition, inhibition of the NF-κB signaling pathway which is critical in M1 macrophage activation was found partly to explain the efficacy of acupuncture treatment in KOA rabbits [35].

The phenotypic polarization of macrophages in the pathogenesis of other locomotory system diseases has also been demonstrated, for example, inflammatory muscle pain [36] and muscle atrophy [37]. In mice with carrageenan-induced inflammatory muscle pain, it was demonstrated that MA at SP6 can relieve nociceptive behaviors including mechanical hyperalgesia, thermalgesia and edema, as well as increasing the IL-10 level in the muscle. The authors also found that MA did not alleviate pain behavior and edema in IL-10 knockout mice. Repeated treatment with SP6 MA could induce the phenotypic transition of muscle macrophages, resulting in decreased numbers of M1 and increased numbers of M2 macrophages, indicating that MA may relieve muscle pain through IL-10-mediated switching of the macrophage phenotype from M1 to M2 [36]. A recent study has also shown that acupuncture plus low-frequency electrical stimulation (Acu-LFES) attenuated denervation-induced muscle atrophy, preventing the loss of soleus and plantaris muscle mass and increasing the muscle cross-sectional area. In addition, Acu-LFES reduced the levels of M1 markers and cytokines (IL-1β, IL-6, IFN-γ and TNF-α) while enhancing the levels of M2 markers and cytokines (Arg-1 and IL-10) [37]. This evidence demonstrates that acupuncture can alleviate the inflammation and pain behavior associated with diseases of the locomotory system, and that the macrophage population and its associated cytokines may be a significant mediator of these therapeutic effects.

### Nervous system

Microglia are macrophage-like immune cells resident in the central nervous system (CNS) and play essential roles in both physiological and pathological conditions. In CNS inflammation, pro-inflammatory factors and neurotoxic molecules which lead to neural network dysfunction and promote inflammatory response are produced by polarized M1 microglia, whereas anti-inflammatory mediators and neurotrophic factors that participate in the restoration of homeostasis can be secreted by polarized M2 microglia. The polarization of microglia has been found to be associated with patient prognosis in neurological diseases, and various molecules have been found to normalize the imbalance between the M1 and M2 phenotypes [38]. For instance, the progression of neurodegeneration in a model of Parkinson’s disease has been found to be related to a gradual increase in neurotoxic pro-inflammatory microglia over the anti-inflammatory phenotype [39].

It has been found that EA decreased the M1-related cytokine IL-1β in the hippocampal CA1 region and sensorimotor cortex in an ischemia–reperfusion injured rat model, along with improvements in motor and memory behavioral performance. This study also highlighted the involvement of p2 purine receptors in microglia/macrophages which were decreased by EA to inhibit neuroinflammation [40]. In similar animal models of stroke, pro-inflammatory cytokines released by M1 macrophages (IL-1β, TNF-α) and activated cellular signaling (NF-κBp65 and p-IκBα) were used as indicators to demonstrate that EA alleviates inflammation in rats with middle cerebral artery occulsion and reperfusion [41]. In spinal cord injury model rats, Zhao et al. [42] found that EA could improve blood–brain barrier function, downregulate M1 macrophage-related factors such as TNF-α, IL-1β, IL-6 and the corresponding surface marker CD86, while M2 macrophage-related factors such as IL-10 and surface marker CD206 were upregulated. Besides acute CNS injuries, EA could alleviate seizures and prevent neuronal death in a mouse model of kainic acid-induced epilepsy, accompanied by inhibiting microglia and astrocyte activation, leading to reduced IL-1β mRNA expression in the hippocampus [43]. Depression is also considered to be related to inflammation, and EA was found to decrease both IL-1β and TNF-α expression, which was also found to alleviate leptin resistance and relieve depression [44]. This evidence indicates that EA can reverse the microglial phenotype to reduce neuroinflammation in CNS injuries.

### Digestive system

The gastrointestinal tract houses the largest total number of macrophages. Besides playing a critical role in pathogen clearance, intestinal macrophages regulate inflammatory responses and local homeostasis [45, 46]. Imbalances in the composition of the intestinal microflora can also trigger pro-inflammatory responses and cause inflammatory bowel disease and other diseases in the host [47]. It has been shown that butyrate reduced the production of pro-inflammatory mediators such as NO and IL-6 in LPS-mediated M1 macrophage activation [48]. It was further shown that defects in M2 macrophage polarization increased the severity of dextran sulfate sodium (DSS)-induced colitis, while the adoptive transfer of M2 macrophages cultured in vitro could reduce the inflammatory reaction in the mouse colon [49, 50]. These results suggest that macrophage polarization is crucial in the prevention and treatment of inflammatory digestive tract diseases such as colitis and peritonitis.

It was found that the M1 macrophage percentage increased while the M2 macrophage percentage decreased in the DSS-induced acute colitis models, and both high-frequency EA (HEA) and low-frequency EA (LEA) at ST36 reduced the protein and mRNA levels of IL-1β, TNF-α, IL-6, IL-12 and IL-17 in the serum and colon, whereas HEA raised the IL-10 level, indicating EA could reverse the M1/M2 ratio. NLRP3/IL-1β protein and mRNA levels in isolated macrophages were further found to be decreased with HEA and LEA, and HEA increased Nrf2/HO-1 levels compared with those in DSS mice, indicating the anti-inflammatory effects of EA on DSS-induced acute colitis may rely on regulating macrophage polarization, NLRP3/IL-1β suppression, and Nrf2/HO-1 promotion [51]. It was also demonstrated that EA had therapeutic effects on trinitrobenzene sulfonic acid (TNBS)-induced colitis, suppressing IL-18 and inducible nitric oxide synthase (iNOS), and increasing IL-10 in the colon [52, 53]. These findings suggest that acupuncture directly or indirectly antagonizes colitis through macrophage polarization in different colitis models.

In acute gastrointestinal diseases, a series of studies have found that acupuncture may improve gastrointestinal symptoms by regulating gastrointestinal macrophages as well. For instance, Yang et al. found that 10 or 30 Hz EA at ST36 up-regulated gastrointestinal motility and attenuated peripheral inflammation in postoperative ileus mice, together with effectively reducing TNF-α and IL-6 in serum. The therapeutic effects on motility and inflammation of 10 Hz EA at ST36 were similar in the mice treated at ST36, ST37, ST39 or CV4, but when applied to ST25, CV12 or non-acupoint had no effect. Moreover, EA at ST36, ST37, ST39 or CV4 inhibited local myeloperoxidase activity and immune cell infiltration, and increased α-smooth muscle actin, indicating that EA at lower limb and abdomen acupoints with the same stimulation parameters had different therapeutic effects on postoperative dysmotility and inflammation [54]. Recently, this group further demonstrated that EA at ST36 suppressed intestinal inflammation and promoted gastrointestinal motility. Mechanistically, EA activated the α7-nicotinic acetylcholine receptor (α7nAChR) -mediated JAK2/STAT3 signaling pathway in macrophages which reduced the production of inflammatory cytokines by acupuncture [55]. In addition, it has been also demonstrated that acupuncture at SP6 has an anti-inflammatory effect on peritonitis caused by carrageenan, resulting in decreased inflammatory cell infiltration and vascular permeability while increasing the levels of IL-10 [56]. They further found that the anti-inflammatory action of SP6 acupuncture depended on both the adrenal glands and increased IL-10 levels. In the sodium-taurocholate-induced severe acute pancreatitis model, the serum concentrations of the M1-related cytokines IL-6 and TNF-α were also decreased after EA treatment [57]. Therefore, it can be seen that ST36 is mostly applyied to inflammatory diseases of the digestive system such as colitis, postoperative ileus, peritonitis and pancreatitis, and the ST36 acupuncture action in regulating gastrointestinal inflammation may be mediated by α7-nicotinic acetylcholine receptor signaling pathways.

### Respiratory system

Asthma is a chronic pulmonary inflammatory disease with high morbidity. Airway hyper-responsiveness and chronic airway inflammation are two key features of asthma and have been shown to be involved in asthma pathogenesis. The phenotypic dysregulation of pulmonary macrophages is considered to be an important factor in asthma pathology [58]. In severe asthma, especially in patients resistant to glucocorticoid therapy, the M1 macrophage phenotype predominates, producing numerous pro-inflammatory mediators, such as TNF-α, IL-1β and NO, which aggravate lung injury and accelerate airway remodeling [59]. Notably, excessive numbers of M2 macrophages may increase cell recruitment and mucus secretion and lead to airway hyperreactivity [60]. Thus, achieving a balance between the M1, M2, and other immunomodulatory macrophages may be the way to achieve the clinical resolution of asthma.

Wei et al. [61] demonstrated that acupuncture at GV14, BL12 and BL13 was effective in the suppression of airway hyper-responsiveness, inhibition of total leukocyte, neutrophil, lymphocyte, and eosinophil counts in bronchoalveolar lavage, as well as TNF-α, IL-1β, IL-5 and eotaxin secretion in the serum of an ovalbumin (OVA)-induced murine asthma model. Moreover, activity in the hypothalamic–pituitary–adrenal (HPA) axis was found to be regulated by acupuncture with the promotion of adrenocorticotropic hormone (ACTH) and cortisol (CORT) secretion in the plasma, enhancing our understanding of the contribution of acupuncture to the regulation of airway inflammation and HPA axis activity in asthma [3]. Another study revealed that the same acupoints applied to acupuncture could alleviate airway hyperresponsiveness and mucus secretion as well as inhibiting NF-κB signaling which may be upstream of the M1-like cytokines in asthmatic mice, accompanied by reductions in the CD4^+^IL-17A^+^ cell numbers, eosinophils, and neutrophils, and increases in the CD4^+^Foxp3^+^ cell numbers in the bronchoalveolar lavage fluid. Furthermore, downregulation of the OVA-specific IgE level and IL-17A, IL-17F and IL-22 levels in the serum was also observed, suggesting that the anti-asthma effect of acupuncture may be associated with inhibition of M1 polarization and regulation of the balance between Th17 and Treg activity [61]. Using the same model, acupuncture at GV14 and bilateral BL13 also upregulated the content of T-box transcription factor (T-bet) and Foxp3^+^, CD4^+^ Foxp3^+^ cell numbers in lung tissue, and the level of IL-10 in the serum. Meanwhile, acupuncture downregulated the RAR-related orphan receptor gamma t level and CD4^+^IL-17A^+^ cell numbers, as well as the serum levels of IL-5, IL-13 and IL-17A [62]. In summary, it can be concluded that the effects of acupuncture on asthma occur mainly through balancing the M1/M2 and Treg/Th17 ratios, indicating a theoretical basis for clinical application using GV14 and BL13 as common acupoints for asthma treatment.

In addition to asthma, the anti-inflammatory action of acupuncture on lung injury also targets macrophage polarization. Limb ischemia/reperfusion can induce inflammation, causing acute lung injury. In a rat model, EA applied at the ST36 and SP6 attenuated lung injury, and decreased the secretion of inflammatory factors such as TNF-α, IL-1, IL-6 and myeloperoxidase, as well as the expression of TLR4 and NF-κB in the lung tissue, indicating that EA can reduce pulmonary inflammation induced by limb I/R injury, possibly via the inhibition of the TLR4/NF-κB pathway [63]. Acupoint catgut embedding (ACE) at ST36 and BL13 also alleviated the respiratory function of rats with LPS-induced acute respiratory distress syndrome, with downregulation of TNF-α and IL-6 levels and upregulation of IL-10 in bronchoalveolar lavage fluid by, indicating that ACE could improve respiratory function by mitigating inflammation by the probable targeting of pro-inflammatory macrophages [64].

Above all, macrophage differentiation is key to the onset and development of disorders in various systems, and acupuncture at different acupoints or combinations of acupoints could regulate macrophage polarization to produce anti-inflammatory effects by regulating cell signaling pathways and cytokine expression and secretion. However, in several systems, direct evidence of macrophage polarization is needed especially future studies focusing on the choice of acupoints that could produce effects on the regulation of cellular differentiation.

## The mechanisms of macrophage polarization regulated by acupuncture

### The upstream regulatory mechanisms of macrophage polarization

Macrophages have different functional characteristics in local microenvironments and present obvious heterogeneity, which are important for the maintenance of homeostasis. Specific organs may alter the macrophage phenotype via the intrinsic and extrinsic factors controlled by tissue microarchitecture, the rate of metabolic activity, or exposure to commensal microorganisms [21]. When challenged, changes in the macrophage signaling pathways lead to alterations in transcription and translation, ultimately resulting in polarization into pro-inflammatory M1 and anti-inflammatory M2 macrophages. For instance, bacterial LPS recognition, cytokines, transcriptional factors, regulators of lipid metabolism, microRNAs (miRNAs), and long non-coding RNAs have been shown in both in vitro and in vivo models to be key regulators of macrophage polarization [19, 65].

Among cytokines, interferons have been recognized to be the prime factors influencing inflammatory macrophages. In an acute peritonitis model, C-terminal truncation of IFN-γ at between Glu135 and Leu136, eliminating the IFN-γ receptor-binding site and thus inactivating the effects of IFN-γ, contributed to the attenuation of pro-inflammatory macrophage activation [66]. Increasing evidence suggests that interferon regulatory factors (IRFs) also play important roles in regulating macrophage functions [67]. Knockout of IRF1 or IRF2 abolished pro-inflammatory responses in murine macrophages in response to LPS or IFN-γ stimulation [68]. Another member of the IRF family, IRF5, was suggested to promote M1 polarization while inhibit M2-associated markers in human peripheral blood macrophages [69], and IRF6 was recently implicated in the negative regulation of M2 polarization of murine bone-marrow-derived macrophage (BMDM) through peroxisome proliferator-activated receptor gamma (PPARγ) inhibition [70]. In contrast, several other IRFs were found to mediate anti-inflammatory type I interferon responses. For example, IRF3 mediated anti-inflammatory signaling and contributed to the M2 activation of human microglia [71] and IRF4 mediated IL-4-induced M2 activation of murine BMDM [72].

Members of the signal transducer and activator of transcription (STAT) family have been identified as key mediators of interferon to prime inflammatory macrophage polarization. STAT1 deficiency in mice abolished the responsiveness of macrophages to IFN-γ and IFN-α. LPS also promoted the formation of STAT1-STAT2 heterodimers that mediate the induction of M1-associated genes through the formation of the IFN-stimulated gene factor 3 complex [73]. In contrast to STAT1/2, STAT6 mediates IL-4a signaling and regulates many M2 signature genes [17]. As a negative regulator of pro-inflammatory genes, PPARγ knockout in myeloid cells was found to reduce M2-like activation and induced susceptibility to obesity, insulin resistance, and glucose intolerance [74], while IL-4 and IL-13 regulated the expression of PPARγ in murine macrophages and human peripheral blood monocytes [75]. Meanwhile, the CCAAT-enhancer-binding protein (C/EBP) family plays an important role in macrophage activation. C/EBPβ mediates the expression of several M2-specific genes, such as the Toll-like receptor-induced expression of Arg-1, mannose receptor c-type 1 (Mrc1), and the macrophage scavenger receptor 1 (MSR1) [76]. Another C/EBP family member, C/EBPσ was shown to induce M1-like pro-inflammatory responses in mouse BMDM [77], while C/EBPα, was required for both M1 and M2 activation of mouse macrophages [78].

There is increasing evidence suggesting that miRNAs regulate macrophage polarization through binding interactions with several key transcription factors. M1 macrophage polarization requires miRNA-125, miRNA-146, miRNA-155, miRNA-let-7a/f and miRNA-378, while M2 polarization requires miRNA-let-7c/e, miRNA-9, miRNA-21, miRNA-146, miRNA-147, miRNA-187 and miRNA-223 [20].

The neural and endocrine systems also participate in the polarization of macrophages. The α7nAChR is an acetylcholine (ACh) receptor expressed on tissue macrophages. Activation of the receptor can inhibit the polarization of M1 induced by LPS and promote the polarization of M2 [79]. Glucocorticoid treatment can increase the percentage of M2 macrophages while decreasing the percentage of M1, and the balance between the two tended to be stable in the early stages of acute lung injury and acute respiratory distress syndrome [80]. Here, we summarize the potential pathways of acupuncture modulation of macrophage polarization via nervous, endocrine and immune regulation.

### Acupuncture induces macrophage polarization through T cells and cytokines

T lymphocyte subsets interact with each other in the normal body to maintain a relatively normal immune function. Typical T helper cells can also polarize, recruit, activate, or differentiate macrophages [81]. Th1, Th2, Th17 and Tregs are subgroups of naive CD4^+^ helper T cells with different functions. During inflammation, Th1 cells produce cytokines such as IFN-γ, TNF-α and granulocyte–macrophage colony-stimulating factor, mediating M1 macrophage polarization, while the Th2 cytokines IL-4 and IL-13 drive macrophage polarization towards the M2 phenotype [65]. The Th17-related cytokine IL-17A can induce increased levels of IL-1, TNF-α and IL-6 [82]. In a mouse model of myocardial infarction, it was found that the increased number of Treg cells led to the polarization of macrophages toward the M2, leading to ventricular remodeling [83]. In the LPS-induced sepsis model, it was shown that induction of M2 macrophages was Treg-dependent [84], which is a strong indication that Treg cells regulate macrophage polarization.

The experimental autoimmune encephalomyelitis (EAE) model is commonly used for the study of multiple sclerosis. Lee et al. found that pretreatment with Bee venom acupuncture (BVA) at ST36 was effective with respect to neurological impairment and loss of body weight in acute EAE rats, and inhibited demyelination and glial activation, suppressed the levels of several pro-inflammatory cytokines and chemokines, including IFN-γ, IL-17, TNF-α, IL-1β, iNOS, RANTES, MCP-1 and macrophage inflammatory protein-1α, and inhibited the activation of the p38 mitogen-activated protein kinase (MAPK) and NF-κBp65 and p-IκBα pathways in the spinal cord of EAE rats. Moreover, pretreatment with BVA also downregulated the number of Th1 and Th17 T cells while upregulating the Treg subpopulation in the spinal cord and lymph nodes, indicating that pretreatment with BVA at ST36 could delay or attenuate the development and progression of EAE by upregulating Treg cells and suppressing Th17 and Th1 responses [85]. In addition, EA may regulate the immune function of OVA-induced delayed-type hypersensitivity (DTH) mice. Wang et al. found levels of Th1 cytokines (IFN-γ and TNF-α) in the footpad tissue were decreased after EA treatment, and IFN-γ-producing CD4^+^ T cells were also inhibited. In addition, the Th1/Th2 ratio was normal after EA treatment [86]. This evidence indicates that acupuncture can promote recovery of the balance between the pro-inflammatory and anti-inflammatory immune cell populations and their associated cytokines.

Our previous study found that the percentage of M1 macrophages in inflamed ankles was decreased, and Treg cells and TGF-β in the lymph nodes were upregulated after acupuncture in AIA rats. The anti-inflammatory effects of acupuncture and inhibition of M1 macrophages in ankles were abolished by TGF-β1-specific receptor blockers. Moreover, acupuncture could inhibit Th1 and Th17 cells in popliteal lymph nodes, upregulate the Treg cell population and related cytokines in murine AIA models, indicating that acupuncture may inhibit pro-inflammatory T cells, and various pro-inflammatory factors in lymph nodes, among which TGF-β secreted by Treg cells is of particular significance in the inhibition of M1 polarization by acupuncture [28]. However, whether acupuncture regulates T cells as an upstream pathway of macrophage polarization needs further confirmation.

Besides T subpopulations and their related cytokines, macrophages are also polarized by themselves and their own related cyokines, such as TNF-α, IL-4, IL-10, IL-13, IL-33 and IL-21 [19]. For instance, Ye et al. found that EA increased the expression of the anti-inflammatory cytokine IL-10 in morphine-induced pruritus model mice, while at the same time promoting M2 macrophage differentiation [87]. It was also found that macrophage-selective deletion of IL-10Rα (IL-10Rα model) markedly intensified DSS-induced colitis and increased pro-inflammatory cytokine production [88], thus, regulating the macrophage-derived IL-10 by EA was speculated to be an upstream modulator of macrophage polarization. However, the mediating roles of other cytokines in macrophage polarization by acupuncture need further investigation.

### Acupuncture regulates macrophage polarization through the nervous system

As a regulatory checkpoint for the immune response, the CNS controls different types of inflammation. Acupuncture induces mechanical stimulation at acupoints that activate afferent nerves that carry information to the somatosensory centers of the CNS. For instance, EA at ST36 activates the nucleus of the tractus solitarius (NTS) through the trigeminal paratrigeminal nucleus of the medulla oblongata. The NTS then coordinates the outgoing pathways directly or indirectly. The direct innervation involves the specific activation of the HPA axis and the parasympathetic nervous system, while the indirect innervation is coordinated by the sympathetic nervous system (SNS) via the cephalic ventrolateral medulla oblongata [89].

The vagus nerve acts as the bridge between the brain and the immune system and is the main component of the parasympathetic branch of the autonomic nervous system (ANS). The immune system activates the vagus sensory fibers of the NTS. After processing, the brain sends out commands via the dorsal motor nucleus (DMN) to activate the efferent fibers of the vagus nerve to control the immune system. Effector neurons of the vagal DMN can inhibit the production of pro-inflammatory cytokines from RTMs [90, 91]. Lim et al. performed acupuncture at ST36 on LPS-induced endotoxemia model mice and found the level of TNF-α was significantly decreased at 30 min and 90 min after acupuncture; in addition, they found that splenic neurectomy and vagotomy resulted in elevated TNF-α signals, which suggests that acupuncture can activate the splenic nerve through the vagus nerve and induce macrophages in the spleen to resist inflammation [92]. Studies have shown that activation of the α7nAChR inhibits the synthesis of pro-inflammatory cytokines and that nanomolar concentrations of ACh are sufficient to inhibit the production of pro-inflammatory cytokines in LPS-induced macrophages [93, 94]. In septic mice models, EA increases the serum levels of dopamine and norepinephrine mediated by vagus nerve stimulation, and dopamine and dopaminergic type-1 (D1) receptor agonists were efficient at inhibiting LPS-induced TNF-α production both in vivo and in vitro, demonstrating that EA can exert anti-inflammatory effects through D1 receptor activation mediated by the vagus nerve [30]. In addition, the dopaminergic type-2 (D2) receptor has also been found to inhibit M1 macrophage differentiation [95], thus the mediating roles of dopamine and its receptors in acupuncture regulation of macrophage polarization needs further investigation.

The SNS, as another part of the autonomic nervous system, plays a bidirectional role in the anti-inflammatory effects of acupuncture. EA at LI4, LI11, Du14 and Du20 of rat forelimbs can activate sensory ganglia and the SNS to mobilize MSCs to enter the systemic circulation, thereby increasing the production of anti-inflammatory cytokines such as IL-10 and promoting tissue repair [96]. It was also found that EA drives sympathetic pathways in somatotopy- and intensity-dependent modes in endotoxin-induced inflammatory models. Low-intensity EA stimulation at the hindlimb ST36 can drive the activation of the vagus-adrenal axis. High-intensity EA stimulation at the abdominal ST25 acupoint can activate the sympathetic axis in the spinal cord subsequently activating neuropeptide Y^+^ splenic noradrenergic neurons, while the two intensity EA both attenuated TNF-α and IL-6 production. They further found that high- and low-intensity EA can activate different adrenergic receptors to produce anti-inflammatory effects [97]. In these models, TNF-α may be secreted from circulating M1 monocytes or tissue-derived macrophages, thus the SNS and its transmitters may mediate macrophage polarization induced by acupuncture.

Apart from the ANS, other neuromodulators are also important in mediating the anti-inflammatory effects of acupuncture. For example, acupuncture can induce the release of 5-hydroxytryptamine, endorphins, and adrenocorticotropin in normal rats to promote the lymphocyte conversion rate [98]. The study found that EA analgesia was blocked by an antibody against β-endorphin and a corticotropin-releasing factor antagonist that were delivered locally in AIA rats [99]. In the same AIA model, EA at GB30 and GB34 elevated the β-endorphin levels in inflamed skin tissue and the percentage of β-endorphin immunoreactive keratinocytes, macrophages, and T-lymphocytes, which suggests that EA induces the release of peripheral opioids at the inflammatory site [100]. Accumulating evidence has indicated that opioids bind to receptors on immune cells to regulate inflammatory cytokines and thus exerting analgesic and immunomodulatory actions. The analgesic effect produced by EA is, therefore, likely to be induced by activating opioid secretion from peripheral inflammatory cells to desensitize peripheral nociceptors and reduce pro-inflammatory cytokines, inhibiting inflammatory pain.

### Acupuncture potentially regulates macrophage polarization through the HPA axis

Dr. Besedovsky first introduced the concept of the "neuroendocrine immunity network" in 1977 [101]. These three systems share signaling molecules, including neuropeptides, neurotransmitters, cytokines, hormones, and their related receptors. The hypothalamus is a major center controlling homeostasis that contains neurons with significant projections to the rest of the limbic region and the sympathetic nucleus, which, in turn, communicate directly with the peripheral nervous system. The hypothalamus, pituitary, and multiple peripheral endocrine glands form a complete endocrine functional system, including the HPA and the hypothalamic-pituitary–gonadal axis. Under stress, corticotropin-releasing hormone (CRH) neurons synthesize and release hormone CRH to the pituitary, stimulating the pituitary to secrete ACTH to act on the adrenal glands, promoting the synthesis and secretion of glucocorticoids. Meanwhile, through feedback regulation of the HPA, glucocorticoids, in turn, regulate the secretion of CRH in the hypothalamic paraventricular hypothalamic nucleus (PVN), reducing the stress response. The release of CRH, ACTH and glucocorticoids through the HPA axis also suppresses the activation of NF-κB signaling in immune cells, thereby reducing the gene expression of the inflammatory cytokines IL-1, TNF-α, IL-6 and IL-8, and stimulating the synthesis of the anti-inflammatory cytokines [102].

It was found that ACTH and β-endorphin concentrations in plasma were increased by EA in deeply anesthetized rats, and c-fos expression was increased in the arcuate nucleus and paraventricular nucleus which contain CRH neurons [103]. Further, EA at GB30 increased the plasma levels of ACTH and corticosterone and activated CRH-containing neurons in the PVN in AIA rats, and pretreatment with CRH and ACTH antagonists prevented the EA-induced anti-edema effect, indicating that HPA axis activation mediates the EA anti-inflammatory action [104]. Similarly, the SP6 and dexamethasone treatments inhibited inflammatory cell infiltration, vascular permeability, and myeloperoxidase activity while increasing IL-10 levels in carrageenan-induced inflammatory pain mice. When the models were adrenalectomized, SP6 acupuncture failed to reduce total leukocyte counts and plasma extravasation [105]. These results imply that EA activates the HPA axis by releasing anti-inflammatory hormones or neuropeptides that target immune organs or damaged tissues. Nevertheless, other studies have shown that EA can prevent raised ACTH and CRH levels in rats after exposure to cold stress. For instance, acupuncture and moxibustion reduced the plasma levels of CORT and ACTH in parturient isolated rats and in a rat model of depression [106, 107]. Thus, the evidence shows that acupuncture has a two-way adjustment effect on the HPA axis in different diseases. In the presence of IL-4 and IL-13, methylprednisolone (MP) increased the CD206^+^ M2a percentage in a dose-dependent way and increased the percentage of CD163^+^ M2c after stimulation by IL-10 and TGF-β, indicating that MP promoted M2a and M2c differentiation [80]. Although in vitro studies have demonstrated that HPA hormones modulate macrophage functions, direct evidence of HPA axis-mediated macrophage polarization induced by acupuncture needs further investigation.

## Conclusion

**Fig. 3:**
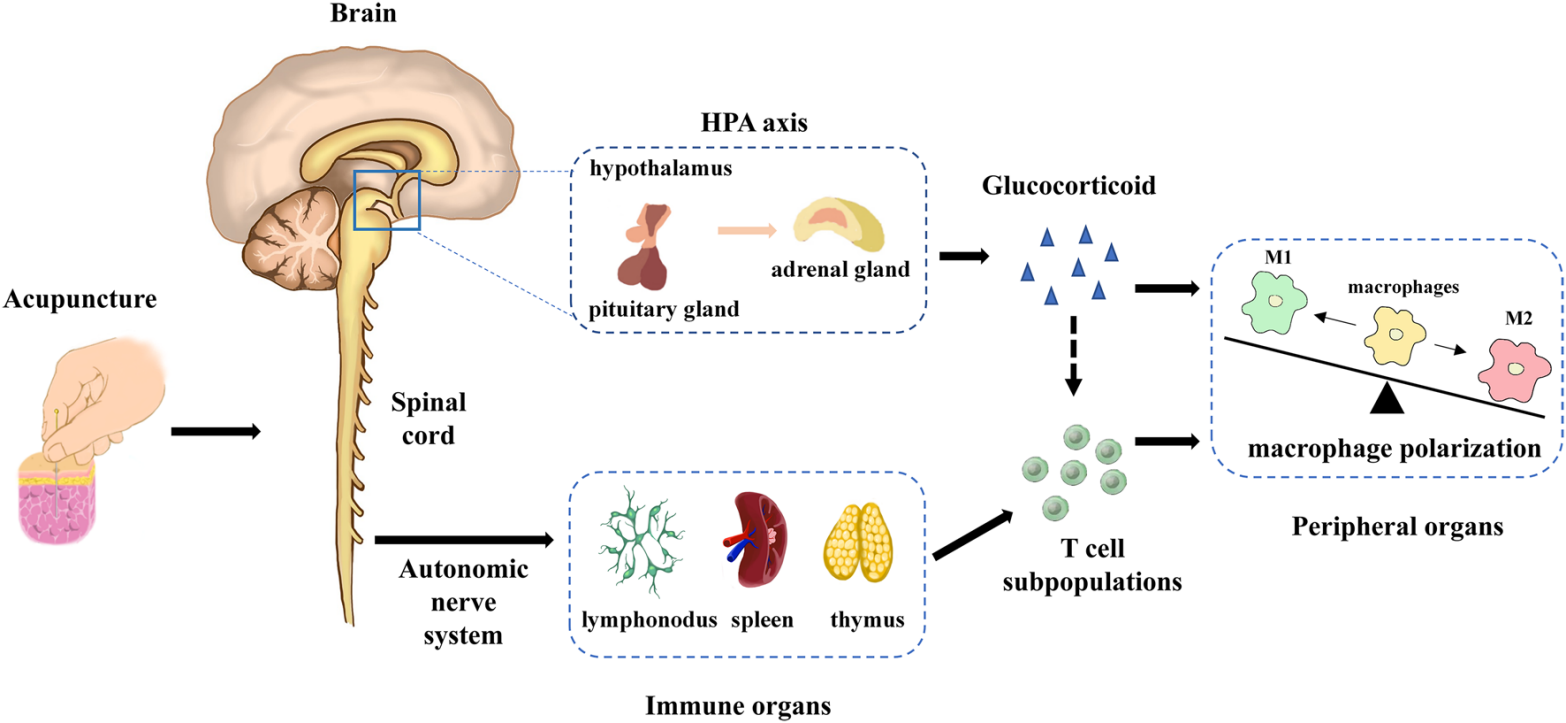
Role of neuroendocrine-immune network regulating macrophage polarization in mediating the anti-inflammatory effects of acupuncture. Acupuncture-induced electrical and biochemical signals within acupoints are transmitted to the central nervous system including spinal cord and brain. Both the hypothalamus–pituitary–adrenal axis and the autonomic nervous system (parasympathetic nerve and sympathetic nerve) are activated, and then glucocorticoids are secreted from adrenal gland and T cell polarization are regulated. Finally, the pro-inflammatory M1 phenotype macrophages in inflammatory organs or tissues are shifted into anti-inflammatory M2 phenotypes, and acupuncture pose anti-inflammation effect

Acupuncture has been widely used in medicine as an effective complementary and alternative therapy in the treatment of many diseases and inflammation. Through a review of the relevant literature, we have found that the anti-inflammatory effects of acupuncture in numerous diseases, including diseases of the immune, locomotory, nervous, digestive and respiratory systems, are closely related to macrophages together with their associated cellular signaling and cytokines. Since specific organs may alter the macrophage phenotype via intrinsic and extrinsic factors, we further conclude that acupuncture can activate the ANS to secrete specific neuromodulators, further inducing macrophage polarization directly or indirectly via regulating T cell polarization and its related cytokines in local inflammatory microenvironments. In addition, acupuncture can promote the release of glucocorticoids and the expression of their receptors in the HPA axis. Finally, the pro-inflammatory M1-phenotype macrophages induced by inflammation in target organs are shifted to anti-inflammatory M2 phenotypes, resulting in the observed anti-inflammatory effect of acupuncture (Fig. 3). Therefore, we speculate that modulation of the nervous-endocrine-immune system leading to the induction of macrophage polarization is an explanation for the effects of acupuncture. This not only increases our understanding of the cellular and molecular mechanisms of acupuncture’s anti-inflammatory action but also focuses on the influence of different systems and their interactions with upstream mechanisms in promoting and enhancing these actions. Nevertheless, there are still many unsolved issues, for instance, the relationship between acupuncture, the neural-endocrine-immune network, and the upstream mechanisms of macrophage polarization which are worthy of further study. Apart from macrophages, other immune cells such as T cells are also the targets of acupuncture, and the interaction between these immune cell populations still needs further investigation.

## Data Availability

Not applicable.

## Abbreviations

RTMs: Resident tissue macrophages
RA: Rheumatoid arthritis
YS: Yolk sac
EMPs: Erythro-myeloid progenitors
Th: T helper cells
IFN-γ: Interferon-gamma
LPS: Lipopolysaccharide
IL: Interleukin
TNF-α: Tumor necrosis factor-α
NO: Nitric oxide
Arg-1: Arginase-1
TGF-β: Transforming growth factor-β
OA: Osteoarthritis
EA: Electroacupuncture
VEGF: Vascular endothelial growth factor
AIA: Adjuvant-induced arthritic
MA: Manual acupuncture
MCP-1: Monocyte chemoattractant protein-1
KOA: Knee osteoarthritis
MSCs: Mesenchymal stem cells
MMP-3: Matrix metalloproteinase-3
Acu-LFES: Acupuncture plus low-frequency electrical stimulation
CNS: Central nervous system
DSS: Dextran sulfate sodium
HEA: High-frequency EA
LEA: Low-frequency EA
TNBS: Trinitrobenzene sulfonic acid
α7nAChR: α7-Nicotinic acetylcholine receptor
iNOS: Inducible nitric oxide synthase
OVA: Ovalbumin
HPA: Hypothalamic–pituitary–adrenal
ACTH: Adrenocorticotropic hormone
CORT: Cortisol
ACE: Acupoint catgut embedding
miRNAs: MicroRNAs
IRFs: Interferon regulatory factors
BMDM: Bone-marrow-derived macrophage
PPARγ: Peroxisome proliferator-activated receptor gamma
STAT: Signal transducer and activator of transcription
C/EBP: CCAAT-enhancer-binding proteins
Mrc1: Mannose receptor c-type 1
MSR1: Macrophage scavenger receptor 1
Ach: Acetylcholine
EAE: Experimental autoimmune encephalomyelitis
BVA: Bee venom acupuncture
MAPK: Mitogen-activated protein kinase
DTH: Delayed-type hypersensitivity
NTS: Nucleus of the tractus solitarius
SNS: Sympathetic nervous system
ANS: Autonomic nervous system
DMN: Dorsal motor nucleus
D1: Dopaminergic type-1
D2: Dopaminergic type-2
CRH: Corticosteroid-releasing hormone
PVN: Paraventricular hypothalamic nucleus
AP: Acute pancreatitis
